# Effect of Grape Over-Ripening and Its Skin Presence on White Wine Alcoholic Fermentation in a Warm Climate Zone

**DOI:** 10.3390/foods10071583

**Published:** 2021-07-07

**Authors:** Pau Sancho-Galán, Antonio Amores-Arrocha, Víctor Palacios, Ana Jiménez-Cantizano

**Affiliations:** Department of Chemical Engineering and Food Technology, Vegetal Production Area, Faculty of Sciences, Agrifood Campus of International Excellence (ceiA3), University of Cadiz, P.O. Box 40, 11510 Puerto Real, Spain; pau.sancho@uca.es (P.S.-G.); victor.palacios@uca.es (V.P.); ana.jimenezcantizano@uca.es (A.J.-C.)

**Keywords:** over-ripening, alcoholic fermentation, white wine, warm climate, yeast, viticulture, climate change

## Abstract

The current trend of rising temperatures and sun irradiation associated to climate change is pushing traditional grape-producing areas with a warm climate towards a very accelerated ripening, leading to earlier harvesting dates and grape must with an unbalanced composition. However, this climatic trend could be exploited to produce other types of wine. In this sense, the increase in temperature could be used to produce wines with overripe grapes. In this regard, the aim of this research work is to evaluate the influence of different degrees and techniques of grape over-ripening to produce wines with the presence or absence of its skins during alcoholic fermentation. To this end, a physicochemical characterization of grape musts and wines obtained from overripe grapes and the monitoring of their fermentation has been performed. Over-ripening grapes by sun-drying has been established as a viable technique viability, producing musts and wines with unique physicochemical and sensory characteristics. In view of the above, it is considered that the production of wines from overripe grapes and in the presence or absence of grape skins is a viable approach to make new white wines taking advantage of the conditions imposed by climate change in a warm climate zone and meet the trends and expectations of current wine consumers.

## 1. Introduction

Grapevines are one of the most important crops worldwide, with vineyard area of 7.4 million hectares and a production of 77.8 million tons of grapes [1]. According to the Food and Agriculture Organization (FAO), in 2018 [2], approximately 37% of world grape production was used for wine production in 71 countries, the 50.7% being produced in three European countries (Italy, France and Spain). These data evidence that the wine industry contributes to the economy and reputation of many countries all over the world. Nowadays, grapevine (*Vitis vinifera* L.) cultivation is primarily located in the Mediterranean basin [3] and other temperate climate regions between the latitudes of 30° and 50° in the northern hemisphere and 40° and 50° in the southern hemisphere [4], although grapevines have been grown outside these limits, in the tropics, for a long time [5]. In overall terms, climate change is gradually modifying the established cultivation limits. More specifically, it is causing a generalized advance of the grape harvest by 10–24 days over the last 30–50 years [6,7,8] and an accelerated vine growth and over-ripening of the grapes, leading to the production of musts with high potential alcoholic strength [9,10], higher pH [11,12], lower acidity [13] and significant nutritional deficiencies, generally resulting in low levels of free amino nitrogen (FAN) [13,14,15]. The effects associated with climate change on grape quality pose important challenges for the winemaking process and the production of quality wines—more particularly, all the factors associated with the expression of varietal aromas, chemical and microbiological stability and sensory balance [7]. Therefore, quality wine production could be affected in those areas that have a warm climate.

To the best of our knowledge, most adaptation actions against climate change focus on the growth and development of different grape varieties [16,17,18,19,20,21]. Among these, the use of later ripening cultivars [22], new and better adapted cultivars [23] and the relocation of vineyards to higher altitudes zones [17] stand out. These adaptation measures would imply a long-term solution and changes in the regulations of the denominations of origin. Therefore, it is considered necessary to continue studying new strategies that will make possible to continue producing quality wines in wine-growing regions, where wine has an important social and economic significance. In this sense, strategies regarding the research of new or better adapted rootstocks [24], irrigation emergency systems [25], protection against extreme heat and sunburns, soil management, changes in training systems [26] and microbiological/biotechnological based strategies [27] are currently investigated all across the world. Regions that already have an eminently warm climate are the ones that are most interested in the search for strategies to adapt to climate change.

For this reason, alongside with the search for adaptation strategies from a viticultural point of view [28,29,30], it is necessary to study new winemaking processes as a strategy for adapting to climate change in particularly warm areas, either by the addition of natural products, to alleviate imbalances in the ripening of the grapes [31,32], or through the search for new winemaking processes [33]. In this sense, one of the strategies to adapt to climate change associated effects could be the elaboration of new white wines from over-ripe grapes. Grape over-ripening is a technique that varies according to climatic conditions and the product to be obtained, as well as the geographical location and the grape variety employed [34]. In China, India and Turkey, it is a method focused on raisin production [35]. However, in most hot and dry countries, this technique has been used for the production of certain sweet and fortified wines.

In this sense, grape over-ripening is a technique that can take advantage of the conditions established by climate change in a warm climate zone (high radiation and temperatures). Thereby, grape over-ripening by means of sunlight techniques allow a natural modification of grape composition and lead to the production of new types of wines. In addition, this adaptation strategy would allow to keep producing wine in traditional winemaking regions, the diversification of its production and the development of new business opportunities. In addition, it would meet the expectations of today’s consumers, eager for oenological concepts in order to recover historical techniques and merge them into new products [35,36].

In view of the above, the aim of this research is to evaluate new white wine production processes that will allow new wines to be made and maintain the continued production of quality white wines in an eminently warm area under the effects of climate change. In this research paper, the results of the production of new dry white wine typologies from the autochthonous grapevine cultivar ‘Palomino Fino’, over-ripped by means of sun-drying and in climatic chamber and also fermented with or without the presence of its skins, are presented.

## 2. Materials and Methods

### 2.1. Raw Material

‘Palomino Fino’ grapes were harvested manually from a vine plot located at 36°64′29.7″ N, 5°49′53.5″ W, at 150 m above sea level, during the two years of study (2018 and 2019). No fertilization or irrigation treatments were applied in the vine plot during the studied years and conventional phytosanitary products were applied to ensure a proper grape development. A control without over-ripening and two different over-ripening techniques were applied to the grapes. On one side, for the sun-drying (SD) technique, grapes were spread out under the sun in a single layer for 48 and 96 h (hence, SD48h and SD96h). On the other side, climatic chamber drying was performed in a drying chamber (CH) (Ibercex ASL, Madrid, Spain), at 35 ± 1 °C and 10% of relative humidity for 48 and 96 h (hence, CH48h and CH96h), in order to compare natural over-ripening versus chamber over-ripening in controlled conditions of temperature and humidity. Temperature and humidity were controlled employing data loggers (LOG-210 Labprocess, Barcelona, Spain) during the whole process.

Once overripe, grapes were destemmed manually and the whole grapes crushed in a vertical press (MECAMAQ M030, Mollerussa, Spain) at a pressure equal to 2 bars. Grape musts were acidified using tartaric acid (Agrovin, Ciudad Real, Spain) and 80 mg/L of potassium metabisulphite (Agrovin, Ciudad Real, Spain). After all the pre-fermentation corrections had been carried out, the different grape musts were distributed in glass-made 5-L tanks. To each tank, displayed in duplicate, an optimal dose of 20% grape skins (GS) calculated by volume was added according with previously published results [33], in order to study their effect on white winemaking. For its fermentation, a *Saccharomyces cerevisiae* pre-ferment of Lalvin 71B^®^ (Lallemand, Barcelona, Spain) was employed. For each vintage studied, the experiment included 10 different fermentations (control without over-ripening, sun-dried and climatic chamber 48 and 96 h) without GS and the same layout with the presence of 20% of GS. The fermentation was carried out under controlled conditions at 18 °C. As soon as the alcoholic fermentation was completed, wines where finned employing gelatin and bentonite at 4 g/hL and 40 g/hL, respectively. After 72 h, wines were filtered by means of a plate filter, bottled, employing nitrogen as inert gas, and corked.

### 2.2. Methodology

Grape must physicochemical characterization (pH, total acidity and °Bé) were performed according to the International Organization of Vine and Wine (OIV) procedures [37]. Free amino nitrogen (FAN) quantification was carried out according to the methodology proposed by Abernathy et al. [38].

Alcoholic fermentation was controlled by a daily measurement of its viable biomass, density and FAN. Viable biomass counts were carried out employing an optical Nikon Microscope using the methylene blue staining method in a Neubauer chamber (Merck, Madrid, Spain). Density was determined in a DMA 5000 M densimeter (Anton Paar, Graz, Austria). Wine analytical measurements (total acidity, volatile acidity and alcoholic strength) were carried out following the methodology stablished by OIV [37]. Residual sugars were determined by means of the dinitrosalicylic acid (DNS) method, according to Gonçalves et al., [39]. CIELab parameters were determined following the recommendations of the International Commission of l’Eclairage [40,41,42]. Absorbance at 420 nm and total polyphenolic index (TPI) were determined using a spectrophotometer Genesis UV-Vis^TM^ 10 s (ThermoScientific, Whaltman, TX, USA), by means of measuring its absorbance at 280 nm wavelength in quartz cuvettes. 

### 2.3. Statistical Analysis

Significant differences between samples were evaluated by two-way ANOVA and Bonferroni multiple range (BSD) test with a *p* < 0.05 (GraphPad Prism version 6.01 for Windows, GraphPad Software, San Diego, CA, USA) statistical package.

## 3. Results and Discussion

### 3.1. Over-Ripening Effects in Grape Must Physicochemical Composition

The results of the physicochemical composition of ‘Palomino Fino’ grape musts after the different grape over-ripening treatments and times during two vintages are shown in Table 1.

For the two vintages studied, the pH values ranged from 3.20 (2018, CH96h) to 3.47 (2018, control). Comparing the different over-ripening treatments, all samples were significantly different from the control (ANOVA *p* < 0.05) during the two vintages studied. The pH values decreased with the hours of over-ripening treatment, this decrease being more pronounced in the case of chamber over-ripening. This may be due to the fact that chamber drying is presented as a continuous process during the treatment, whereas sun drying is paused at night. Closely related to pH, the total acidity values of the samples showed a similar trend, increasing with the treatment hours, from 3.62 g/L for control (2019) to 5.78 g/L of tartaric acid for CH96h (2019), this increase being more remarkable and significantly different (ANOVA *p* < 0.05) than the rest of the samples for climatic chamber drying (both, 48 and 96 h). The increase in total acidity values depends on the difference between the acid concentration phenomenon due to the evaporation of the vegetation water present in the grapes [43] and the metabolism of malic acid by respiratory combustion [44]. As can be seen in Table 1, the acidity increases that occurred in the sun-dried grapes as well as in the chamber dried grapes during the 2018 campaign are much lower than those corresponding to the 2019 campaign, probably due to a higher consumption of malic acid. However, in all cases, the pH variation has been much smaller than in the case of acidity, possibly due to the difficulty of altering a buffered medium such as wine [45]. The FAN content shows a similar behavior for the two vintages studied, increasing with the time of over-ripening and being significantly higher (ANOVA *p* < 0.05) in the case of over-ripening in climatic chamber. Grape musts from the 2019 vintage showed a higher FAN content compared to 2018, possibly due to concentration phenomena. Increasing the FAN content in musts has benefits such as improving yeast cell growth at the beginning and during alcoholic fermentation [46,47], as well as an increased survival of yeasts at the end of alcoholic fermentation [48]. This FAN increase is positive in order to improve the fermentative potential of grape musts with deficiencies that could lead to stuck or sluggish fermentations due to its high sugar content [49]. 

### 3.2. Effect of Over-Ripening and Grape Skin (GS) Presence during Alcoholic Fermentation

**Figure 1:**
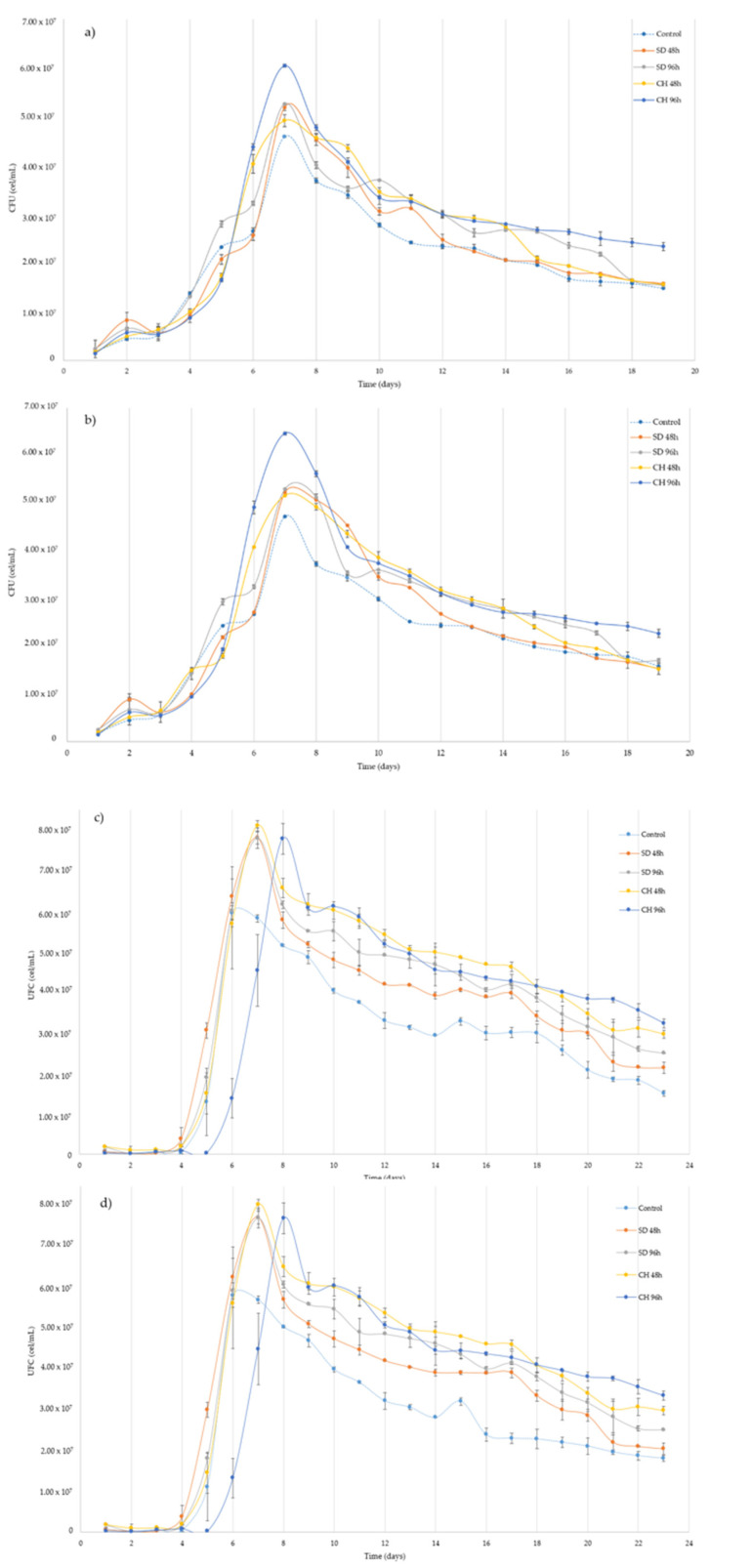
(**a**–**d**) Viable biomass development during grape must alcoholic fermentation without (**a**,**c**) and with (**b**,**d**) the presence of grape skins during two vintages (2018, **a**,**b**, and 2019, **c**,**d**). Control: without over-ripening. SD48h: sun-dried grapes during 48 h. SD96h: sun-dried grapes during 96 h. CH48h: climatic chamber drying during 48 h. CH96h: climatic chamber drying during 96 h.

Figure 1a–d show the evolution of the viable yeast population during the alcoholic fermentation process during the vintages 2018 and 2019 with GS (b,d) and control (a,c), respectively.

Different grape over-ripening treatments, as well as fermentation with or without the presence of skins, do not affect the yeast lag phase times during the alcoholic fermentation. Year-on-year, the lag phase in the 2019 vintage is slightly longer, possibly due to the higher concentration of present sugars in the medium and, therefore, a greater osmotic shock and difficulty for the yeasts to adapt to the fermentation medium. For all the cases studied, the exponential phase begins 72–96 h after the yeast inoculation, reaching the maximum population after 7 days for all cases in the 2018 vintage and between days 6 and 8 for the 2019 vintage, depending on the time and over-ripening technique used. Regarding the maximum populations reached, in all cases of study the populations are significantly higher than those presented by the control (ANOVA *p* < 0.05), the CH96h samples being the ones with a greater yeast population in the 2018 vintage and the CH48h samples in the 2019 vintage. Once the maximum populations were reached, a progressive decrease in yeast populations was observed for both over-ripening techniques, regardless of the presence or absence of skins (ANOVA *p* < 0.05). It can be observed that the final yeast populations show a higher survival in the case of a higher concentration of sugars at the beginning of fermentation, thus confirming the positive correlation between yeast survival time and the concentration of sugars present in the medium [50]. Similarly, these results coincide with those that state that musts rich in sugars carry out a large part of the alcoholic fermentation with their yeasts in the decline phase [51]. Alcoholic fermentation is extended by 19 days for the 2018 vintage and 23 days for the 2019 vintage, possibly due to a higher initial concentration of sugars in the latter case.

Comparing the presence or absence of 20% of GS during alcoholic fermentation, a slight increase in yeast populations was observed in the cases where the musts had GS, without affecting the different stages of yeast growth or the time necessary to carry out fermentation. In this sense, control wines with GS presence showed a 27.7% and 25.1% higher population for the 2018 and 2019 vintages, respectively. In the same way and coinciding with the results of recently published research papers, it is confirmed that the presence of a certain amount of GS is able to sponsor a greater growth and survival of yeasts [33]. This may possibly be due to the presence of nitrogen compounds and other growth cofactors necessary for yeast growth found in grape skins [52,53]. Thus, the presence of a 20% of GS ensures a higher population of viable yeasts in the fermenters, regardless of the grape over-ripening time and technique. 

### 3.3. Over-Ripening and GS Presence Effect on the Alcoholic Fermentation kinetics

**Figure 2:**
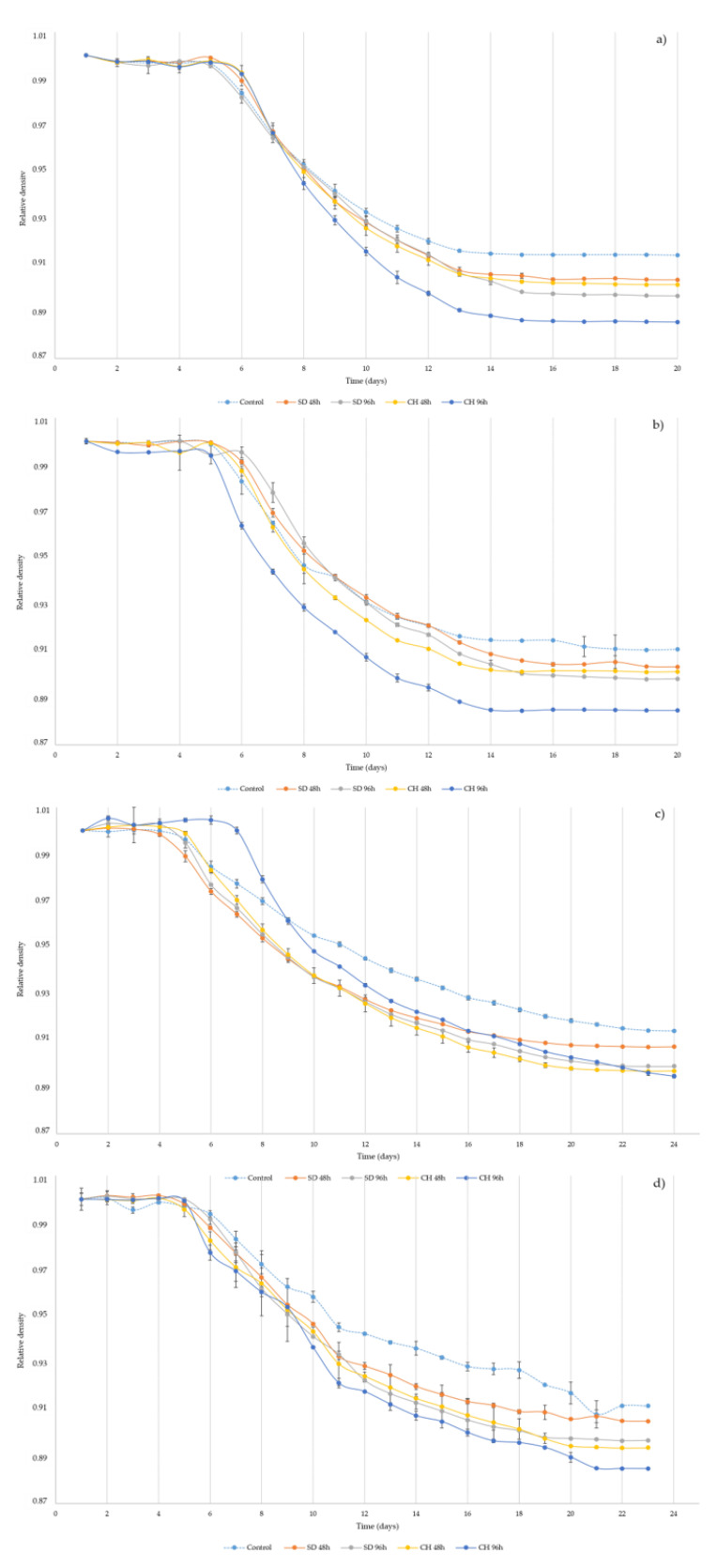
(**a**–**d**) Relative density evolution during grape must alcoholic fermentation without (**a**,**c**) and with (**b,d**) the presence of grape skins during two vintages (2018, **a**,**b,** and 2019, **c**,**d**). Control: without over-ripening. SD48h: sun-dried grapes during 48 h. SD96h: sun-dried grapes during 96 h. CH48h: climatic chamber drying during 48 h. CH96h: climatic chamber drying during 96 h.

Figure 2a–d show the evolution of the relative density in the different alcoholic fermentation processes during the vintages 2018 and 2019 with GS (b,d) and control (a,c). 

At the beginning, fermentation is slow with no significant variations in the density of the different samples until the fifth day of fermentation, regardless of the over-ripening technique, the time of over-ripening and the presence or absence of GS in the fermentation medium. Once the exponential yeast growth starts, the decrease in density starts to accelerate. During the days when the yeast has a higher reproduction rate, a greater decrease in relative density is observed. Year-on-year, samples from the 2018 vintage (Figure 2a,b) show a higher fermentation rate than those from the 2019 vintage (Figure 2c,d). As mentioned before, higher sugar concentrations could again be responsible for the slightly slower beginning of fermentation, especially in the case of sample CH96h 2019 (Figure 2c). However, the concentration of compounds by means of water evaporation in grapes during the over-ripening process implies a higher concentration of compounds necessary for yeasts, such as nitrogenized compounds (Table 1), and, therefore, its extensive development (Figure 2c) [54]. As expected, a higher viable biomass implies, in all cases, a higher fermentation speed given the higher consumption of sugars by yeasts. In all cases, it is observed that control samples, made with grapes without over-ripening, are the first to show a slowdown in their fermentation speed, showing a significantly higher relative density at the end of fermentation (ANOVA *p* < 0.05). This fact is supported by the observations in Figure 1, where it can be seen that a lower viable biomass implies a slower fermentation rate in the final phase of alcoholic fermentation. As for the contribution of 20% GS (Figure 2b,d), this practice implies an extra contribution of nutrients and/or co-factors, such as minerals and vitamins from the grape skins (Figure 2b,d) [52,53]. However, significant differences (ANOVA *p* < 0.05) were only observed for the CH96h samples, where the fermentation rate was higher. Nevertheless, for the rest of the samples, no major differences in fermentation kinetics were detected, coinciding with the recent results of the research group, a higher proportion of GS being necessary to significantly accelerate the fermentation process [33]. Finally, none of the samples during the two vintages studied presented problems in the final phase of fermentation, which could be carried out correctly until the end.

### 3.4. Over-Ripening and GS Presence Effect in Free Amino Nitrogen (FAN) during Alcoholic Fermentation

**Figure 3:**
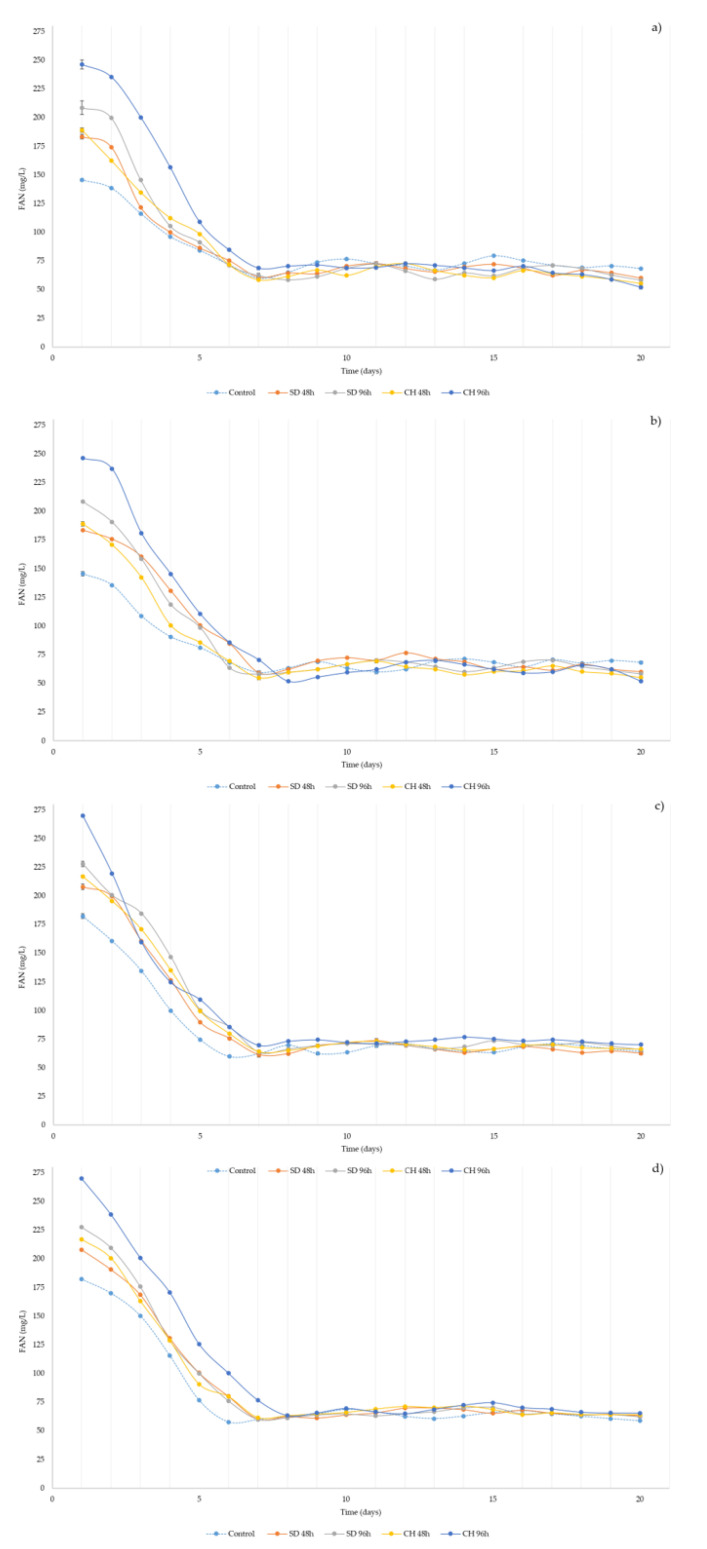
(**a–d**) Free amino nitrogen (FAN) concentration during grape must alcoholic fermentation without (**a**,**c**) and with (**b**,**d**) the presence of grape skins during two vintages (2018, **a**,**b,** and 2019, **c**,**d**). Control: without over-ripening. SD48h: sun-dried grapes during 48 h. SD96h: sun-dried grapes during 96 h. CH48h: climatic chamber drying during 48 h. CH96h: climatic chamber drying during 96 h.

Nitrogenized compounds are necessary for the proper development of yeasts and alcoholic fermentation, nitrogen being the most important compound in fermentation, after carbon. FAN represents those nitrogenous compounds that are available to yeast, i.e., amino acids and ammonium, as well as some small peptides [38]. Figure 3a–d show the evolution of FAN content during alcoholic fermentation for the different over-ripening times and techniques, as well as for the presence or absence of skins in the fermentation medium.

Grape over-ripening implies an increase in FAN concentration, with maximum values for the CH96h samples and minimum values for the control samples that did not receive any over-ripening treatment during the two years of study. At the beginning of fermentation, all samples showed significant differences among them (ANOVA *p* < 0.05). Unlike what was observed for viable biomass (Figure 1) and relative density (Figure 2), FAN content starts to decrease after the first 24 h from the inoculation of the yeasts in the fermentation medium. This is due to the consumption of substances nitrogenized by the yeasts for their survival and reproduction. The minimum values of FAN were obtained between days 6 and 7 of fermentation, coinciding, in all cases, with the highest values of viable biomass present in the fermentation tanks and the moment when yeast began to consume more sugars, thus, a higher fermentation activity. Once the minimum FAN content was reached, it was observed that the concentrations of this parameter showed an oscillatory nature until the end of fermentation, with significant differences being observed only occasionally and in some cases in the overripe samples, with and without skins, with respect to the control (ANOVA *p* < 0.05). In this case, the addition of 20% of skins to the fermentation medium led to a less pronounced decrease in the FAN content in all the samples for the two vintages studied, which could be due to the extra release of nitrogenized compounds from GS into the fermentation medium.

For all the samples and during the two vintages analyzed, FAN content was above 140 mg/L in grape musts, which is the limit defined for a correct alcoholic fermentation by the yeasts [55]. Once most of the FAN was consumed, the oscillatory nature observed in all samples on an inter-annual basis may have been due to the autolysis process of the dead yeasts, which release, into the environment, compounds considered to be part of the FAN, such as some amino acids [56,57,58,59]. The final FAN values in the samples were significantly higher for the 2018 vintage, compared to 2019; nevertheless, the final values in all samples and for both vintages ensured that wines were stable from a microbiological point of view, as well as minimizing the occurrence of other problems, such as the accumulation of harmful compounds in the wine, such as ethyl carbamate [32,33].

### 3.5. Effect of Over-Ripening and the Presence of GS on the Physicochemical Composition of Final Wines

Table 2 shows the physicochemical characterization of the final wines made from overripe grapes and in the absence or presence of GS.

As for total acidity, wines from the 2019 vintage showed higher values of total acidity than those made in the 2018 vintage, as was also the case for grape musts. More specifically, it can be seen that the results fluctuate in one way or another depending on the over-ripening technique and its time of application. Thus, the wines made by overripe grapes in a climatic chamber for 96 h had the highest total acidity values, in all cases, and were significantly different (ANOVA *p* < 0.05) compared to the control, which presented the lowest values, in all cases. On the other hand, the presence of GS in the fermentation medium caused a slight decrease in wine acidity, which could be due to the release of minerals, such as potassium, by GS [60], resulting in tartaric precipitation. Such decrease has been previously observed by Olejar et al. [61] in white wines made from ‘Sauvignon Blanc’. In general, the increase of total acidity in wines made from over-ripened grapes is mainly due to an increase in the concentration, explained by the decrease in the amount of water [62]. As for volatile acidity, there is a clear tendency in this parameter to increase with both the over-ripening time and technique, being higher in climatic chamber over-ripening than in sun-drying. In general, the proportion of acetic acid produced during fermentation increases proportionally with the initial sugar concentration. The minimum value of volatile acidity corresponds to the control wine in all cases and the highest value for the CH96h wine, with significant differences between them (ANOVA *p* < 0.05) during the two vintages studied. As for the presence of grape skins, it is observed that their contribution implies an increase in the volatile acidity of the final wines. This volatile acidity increase in wines made in the presence of GS could be due to an increase in the concentration of volatile acid esters and alcohols, whose concentration in wines depends on the maceration time [63]. Acetic acid is the main component of volatile acidity and has a major influence on wine quality [64]. Acetic acid concentration in dry wines normally ranges between 0.1 and 0.5 g/L and its threshold of perception varies in the range of 0.7–1.0 g/L [65]. In winemaking from sound grapes, this acid is produced by yeasts during alcoholic fermentation, in different concentrations depending on the species and strain of yeast used [66], although it can also be synthesized by lactic and acetic acid bacteria [58]. On the other hand, under the osmotic stress conditions to which yeasts are subjected in musts with high sugar concentration, they express genes that regulate glycolysis and the pentose phosphate pathway, thereby increasing the synthesis of fermentation by-products, such as glycerol and acetic acid [67]. However, there is also the possibility that some of the acetic acid is of accidental origin, due to the presence of lactic acid bacteria in the grape must in the grapes [58].

Logically, as with the sugar concentration in the initial musts (Table 1), wines made from overripe grapes showed higher alcohol content values than the control. Specifically, the wines made from musts with higher sugar content provided wines with higher alcohol content. This is the case of CH96h, which showed significantly higher values (ANOVA *p* < 0.05) than the rest of the wines. In this case, the addition of 20% of GS did not influence the alcohol content of final wines, coinciding with the results recently published [33]. During the alcoholic fermentation metabolic pathway, sugar in grape must is transformed into ethanol and other by-products by fermenting yeasts. However, yeasts are not tolerant to high alcoholic strength. In this sense, yeasts tolerance to ethanol can be observed in the results of residual sugar analysis. Those wines that have shown a higher alcoholic content have also presented higher residual sugar values at the end of fermentation. Thus, for the two vintages studied, wines made from overripe grapes for 96h in the climatic chamber showed significantly higher residual sugar values (ANOVA *p* < 0.05) than the control and some samples. From a certain alcoholic strength onwards, cell viability starts to decrease, due to the stress to which the yeast is subjected by ethanol. This decrease in cell viability starts to be observed from 13% *v/v* in *Saccharomyces cerevisiae* [67]. Ethanol tolerance is a factor that can lead to incomplete fermentation. The toxicity of ethanol arises due to its ability to interact with membranes, altering their fluidity and, as a consequence, all metabolic functions of the cell [68]. As for the polyphenolic content present in final wines (TPI), the ones from the 2018 vintage have higher values than those from the 2019 vintage, which may be due to the greater polyphenolic maturity of the grapes at the time of harvest. However, the over-ripening process of these grapes implies an increase in the TPI values, with the maximum values again being observed for the CH96h samples. White grapes over-ripening causes a series of changes in the phenolic composition of wine, resulting in browning as a result of the formation of brown pigments due to the Maillard reaction [69]. This process is favored by large quantities of sugars, the high temperatures reached by the grapes during over-ripening and the polymerization of phenolic compounds [70]. However, some oxidative phenomena may occur due to the high temperatures and the degree of insolation reached during the over-ripening process, leading to a reduction of the polyphenol content in grapes skins and must [71,72,73]; consequently, the decrease in TPI values for SD48h and SD96h in both vintages when fermented without GS with respect to the control can be explained. On the other hand, wines made with 20% of GS show slightly higher data than wines made conventionally, which shows a transfer of compounds present in the GS during alcoholic fermentation [74].

The absorbance at 420 nm of a wine gives an indication of the yellow color that white wines should have. This wine quality control parameter is associated with the presence of yellowish-brown pigments and, therefore, measures the rate of oxidation of polyphenols in white wine [69]. The absorbance at 420 nm values for the different samples shows significant differences from a statistical point of view, but they are almost negligible from an oenological point of view. It can be seen that the wines made by chamber over-ripened grapes have higher values than those made by sun-drying. However, it is noteworthy that, in wines made in the presence of GS, the behavior of the values has been the opposite. Control wines showed significantly higher browning values than those made from overripe grapes (ANOVA *p* < 0.05), regardless the technique employed. In this sense, the presence of skins during winemaking could exert a protective effect against possible oxidation effects during alcoholic fermentation [33]. For wine color, CIELab is the L*a*b* color space, which is one of the most popular color spaces for measuring samples. In CIELab, L* indicates brightness or lightness, varying in value between 0, which indicates black, or minimum brightness, and 100 which relates to white, or maximum brightness. On the other hand, a* and b* are the chromatic coordinates [75]. In terms of brightness, all wines showed very similar values, with no significant differences between them. All the values are close to 100, which indicates that they are bright and luminous wines. In regard to the chromatic coordinates, significant differences were observed between some wines, mainly between the control wine and the rest, and also between the wines with different over-ripening techniques and/or time.. If we look at Table 2, all the wines show values close to zero for a* and positive values for b*, which means that all of them, to a greater or lesser extent, have a yellow-greenish color. Finally, as for the hue angle, no significant differences were observed between the different wines, all of which were close to 100, indicating a high hue.

## 4. Conclusions

In conclusion, grape over-ripening increases the sugar content and other organic compounds, such as acids and FAN, in the initial grape musts. The start of fermentation was more difficult the higher the grape over-ripening degree was and, therefore, the greater the concentration of sugars in its must. In terms of fermentation kinetics, over-ripening did not influence yeast lag phase; however, the presence of 20% of GS sponsored a higher yeast population. Final wines showed significant differences depending on the time and technique of over-ripening used. From a physico-chemical point of view, the presence of GS slightly increases the volatile acidity and TPI, but on the other hand decreases the degree of browning. Nevertheless, in all cases, it has been possible to produce dry white wines without problems at any stage of fermentation or deviations in their basic oenological or chromatic parameters. Comparing the two techniques, it has been observed that sun-drying is capable of producing significant modifications in wines, diversifying production and having lower requirements in terms of facilities, investment and energy, than the drying procedure in a climatic chamber. With the intention of further deepening this research, it would be advisable to carry out this study for more vintages, in order to check the reproducibility of the technique, as well as to try it with other white wine varieties. In view of the above, it is considered that the production of wines from overripe grapes and in the presence or absence of GS is a viable approach to make new white wines taking advantage of the conditions imposed by climate change in a warm climate zone and meet the trends and expectations of current wine consumers.

## Figures and Tables

**Table 1:** Grape must physicochemical composition after grape over-ripening treatments.

	Control	SD48h	SD96h	CH48h	CH96h
	2018				
pH	3.470 ± 0.014 ^a^	3.440 ± 0.014 ^b^	3.420 ± 0.028 ^c^	3.300 ± 0.014 ^d^	3.200 ± 0.014 ^e^
TA (g/L)	3.630 ± 0.117 ^a^	3.640 ± 0.175 ^a^	3.920 ± 0.058 ^b^	4.106 ± 0.058 ^c^	4.996 ± 0.058 ^d^
FAN (mg/L)	145.600 ± 0.000 ^a^	183.400 ± 1.980 ^b^	208.600 ± 5.940 ^c^	189.000 ± 1.980 ^b^	246.400 ± 3.960 ^d^
°Bé	11.300 ± 0.140 ^a^	12.800 ± 0.140 ^b^	13.500 ± 0.140 ^c^	12.800 ± 0.000 ^b^	15.000 ± 0.140 ^d^
	2019				
pH	3.360 ± 0.021 ^a^	3.290 ± 0.042 ^b^	3.230 ± 0.035 ^c^	3.280 ± 0.078 ^b^	3.230 ± 0.070 ^c^
TA	3.620 ± 0.080 ^a^	4.310 ± 0.053 ^b^	5.525 ± 0.053 ^c^	5.063 ± 0.043 ^d^	5.780 ± 0.070 ^e^
FAN (mg/L)	162.500 ± 2.256 ^a^	200.230 ± 1.978 ^b^	224.600 ± 1.450 ^c^	207.650 ± 2.465 ^b^	265.130 ± 3.472 ^d^
°Bé	12.180 ± 0.020 ^a^	12.770 ± 0.040 ^b^	13.910 ± 0.090 ^c^	14.210 ± 0.060d ^c^	15.680 ± 0.030 ^e^

Control: without over-ripening. SD48h: sun-dried grapes during 48 h. SD96h: sun-dried grapes during 96 h. CH48h: climatic chamber drying during 48 h. CH96h: climatic chamber drying during 96 h. FAN: free amino nitrogen. TA: total acidity (g/L tartaric acid). °Bé: Baumé degrees. Different superscript letters mean a significant difference between samples (ANOVA *p* < 0.05) determined by two-way ANOVA applying a Bonferroni multiple range (BSD) test.

**Table 2:** Wine physicochemical and color characterization.

	**2018**				
	**Control**	**SD48h**	**SD96h**	**CH48h**	**CH96h**
	Without GS				
TA (g/L)	4.629 ± 0.027 ^a^	4.763 ± 0.067 ^a^	4.905 ± 0.080 ^a^	5.102 ± 0.241 ^b^	5.554 ± 0.013 ^b^
VA (g/L)	0.162 ± 0.012 ^a^	0.184 ± 0.031 ^a^	0.400 ± 0.024 ^b^	0.231 ± 0.012 ^a^	0.366 ± 0.021 ^b^
% Alc.	11.854 ± 0.182 ^a^	13.430 ± 0.060 ^a^	14.633 ± 0.159 ^a,b^	13.656 ± 0.398 ^a^	16.584 ± 0.016 ^c^
RS (g/L)	1.418 ± 0.285 ^a^	1.922 ± 0.330 ^b^	2.220 ± 0.509 ^b^	1.733 ± 0.107 ^a,b^	2.998 ± 0.264 ^c^
TPI	7.990 ± 0.141 ^a^	6.540 ± 0.170 ^b^	6.090 ± 0.269 ^b^	6.450 ± 0.891 ^b^	8.160 ± 0.085 ^a^
Abs 420	0.074 ± 0.015 ^a^	0.093 ± 0.008 ^a^	0.093 ± 0.001 ^a^	0.109 ± 0.008 ^b^	0.110 ± 0.002 ^b^
L*	96.834 ± 0.923 ^a^	98.279 ± 0.057 ^a^	98.664 ± 0.173 ^a^	98.372 ± 0.071 ^a^	98.247 ± 0.127 ^a^
a*	0.160 ± 0.119 ^a^	0.420 ± 0.020 ^b^	0.470 ± 0.033 ^b^	0.600 ± 0.064 ^c^	0.430 ± 0.023 ^b^
b*	10.484 ± 2.874 ^a^	5.120 ± 0.572 ^b^	4.860 ± 0.246 ^b^	5.683 ± 0.492 ^b^	6.168 ± 0.009 ^b^
H*	91.019 ± 0.931 ^a^	94.671 ± 0.301 ^a^	95.526 ± 0.664 ^a^	96.021 ± 0.115 ^a^	93.970 ± 0.217 ^a^
	With 20% GS				
TA (g/L)	4.413 ± 0.102 ^a^	4.569 ± 0.140 ^a,b^	4.769 ± 0.097 ^b^	4.958 ± 0.305 ^b,c^	5.068 ± 0.198 ^c^
VA (g/L)	0.361 ± 0.068 ^a^	0.412 ± 0.006 ^a,c^	0.580 ± 0.100 ^b^	0.428 ± 0.168 ^c,d^	0.551 ± 0.136 ^b,d^
% Alc.	11.970 ± 0.256 ^a^	13.569 ± 0.147 ^a,b^	14.896 ± 0.253 ^a,b^	13.852 ± 0.539 ^a,b^	16.489 ± 0.187 ^c^
RS (g/L)	1.257 ± 0.149 ^a^	1.567 ± 0.698 ^a^	2.541 ± 0.410 ^b^	1.710 ± 0.205 ^a^	3.056 ± 0.423 ^b^
TPI	8.690 ± 0.157 ^a^	7.214 ± 0.099 ^a^	7.724 ± 0.301 ^a^	7.158 ± 0.249 ^a^	8.879 ± 3.265 ^a^
Abs 420	0.158 ± 0.008 ^a^	0.087 ± 0.005 ^b^	0.048 ± 0.001 ^c^	0.087 ± 0.003 ^b^	0.099 ± 0.001 ^b^
L*	98.698 ± 0.587 ^a^	100.025 ± 0.147 ^a^	101.259 ± 0.257 ^a^	99.995 ± 0.009 ^a^	102.025 ± 0.298 ^a^
a*	0.153 ± 0.111 ^a^	0.411 ± 0.015 ^b^	0.468 ± 0.019 ^b,c^	0.530 ± 0.054 ^c^	0.384 ± 0.069 ^b^
b*	5.699 ± 0.547 ^a^	3.568 ± 0.413 ^b^	2.567 ± 0.154 ^b^	2.541 ± 0.056 ^b^	2.354 ± 0.016 ^b^
H*	93.545 ± 0.931 ^a^	97.541 ± 0.149 ^a^	98.035 ± 0.761 ^a^	98.221 ± 0.431 ^a^	96.028 ± 0.199 ^a^
	**2019**				
	**Control**	**SD 48 h**	**SD 96 h**	**CH 48 h**	**CH 96 h**
	Without GS				
TA (g/L)	5.570 ± 0.098 ^a^	5.810 ± 0.104 ^a^	6.480 ± 0.057 ^b^	6.320 ± 0.421 ^b^	6.460 ± 0.268 ^b^
VA (g/L)	0.189 ± 0.030 ^a^	0.214 ± 0.012 ^a^	0.256 ± 0.036 ^b^	0.296 ± 0.016 ^b^	0.489 ± 0.080 ^c^
% Alc.	10.756 ± 0.430 ^a^	12.380 ± 0.320 ^a,d^	14.299 ± 0.190 ^b^	13.420 ± 0.598 ^b,d^	16.240 ± 0.480 ^c^
RS (g/L)	1.356 ± 0.018 ^a^	1.976 ± 0.143 ^b^	1.447 ± 0.169 ^a,b^	1.238 ± 0.188 ^a^	4.813 ± 0.268 ^c^
TPI	6.513 ± 0.091 ^a^	4.976 ± 0.100 ^b^	3.790 ± 0.082 ^c^	5.713 ± 0.712 ^b^	10.268 ± 0.55 ^d^
Abs 420	0.040 ± 0.010 ^a^	0.051 ± 0.010 ^a,d^	0.062 ± 0.001 ^b,d^	0.073 ± 0.010 ^b^	0.110 ± 0.01 ^c^
L*	97.563 ± 1.235 ^a^	98.593 ± 0.147 ^a^	97.305 ± 0.846 ^a^	95.168 ± 0.992 ^a^	96.436 ± 0.589 ^a^
a*	0.5 ± 0.006 ^a^	0.769 ± 0.110 ^b^	0.782 ± 0.015 ^b^	0.988 ± 0.036 ^c^	0.846 ± 0.087 ^b^
b*	10.312 ± 0.653 ^a^	11.241 ± 0.216 ^a^	11.983 ± 0.549 ^a^	10.673 ± 0.630 ^a^	14.297 ± 0.55 ^b^
H*	97.536 ± 2.541 ^a^	98.631 ± 0.964 ^a^	97.995 ± 0.966 ^a^	104.531 ± 2.174 ^a^	93.631 ± 0.501 ^a^
	With 20% GS				
TA (g/L)	5.170 ± 0.070 ^a^	5.460 ± 0.070 ^b^	6.118 ± 0.050 ^c^	6.025 ± 0.560 ^c^	6.165 ± 0.150 ^c^
VA (g/L)	0.230 ± 0.030 ^a^	0.240 ± 0.010 ^a,b^	0.280 ± 0.010 ^b^	0.340 ± 0.010 ^c^	0.620 ± 0.030 ^d^
% Alc.	10.860 ± 0.910 ^a^	12.430 ± 0.440 ^b^	14.390 ± 0.260 ^c^	13.390 ± 1.470 ^b,c^	16.480 ± 0.360 ^d^
RS (g/L)	1.200 ± 0.050 ^a^	1.450 ± 0.090 ^a,b^	1.640 ± 0.001 ^a,b^	1.770 ± 0.140 ^b^	4.510 ± 0.080 ^c^
TPI	4.320 ± 0.160 ^a^	5.620 ± 0.100 ^b^	7.260 ± 0.070 ^c^	6.350 ± 0.880 ^b,c^	10.974 ± 0.550 ^d^
Abs 420	0.140 ± 0.010 ^a^	0.058 ± 0.010 ^b^	0.063 ± 0.001 ^b^	0.071 ± 0.010 ^b^	0.780 ± 0.010 ^b^
L*	100.220 ± 0.430 ^a^	100.110 ± 0.050 ^a^	100.190 ± 0.000 ^a^	97.990 ± 2.020 ^a^	98.900 ± 0.140 ^a^
a*	0.511 ± 0.130 ^a^	0.862 ± 0.090 ^b^	0.852 ± 0.010 ^b^	1.053 ± 0.250 ^c^	0.911 ± 0.030 ^b,c^
b*	3.330 ± 0.300 ^a^	4.280 ± 0.640 ^a,b^	4.780 ± 0.010 ^a,b^	3.660 ± 0.490 ^a^	9.900 ± 0.550 ^b^
H*	100.290 ± 1.810 ^a^	101.480 ± 1.220 ^a^	100.100 ± 0.130 ^a^	106.480 ± 5.660 ^a^	95.270 ± 0.490 ^a^

SD48h: sun-dried grapes during 48h. SD96h: sun-dried grapes during 96h. CH48h: climatic chamber drying during 48 h. CH96h: climatic chamber drying during 96 h. TA: total acidity (g/L tartaric acid). VA: volatile acidity (g/L acetic acid). RS: residual sugars. TPI: total polyphenolic index. CIELab coordinates: L* (lightness), a* (red/green), b* (yellow/blue), H* (hue) and C* (chroma). Different superscript letters mean a significant difference between the samples (ANOVA *p* < 0.05) determined by two-way ANOVA applying a Bonferroni multiple range (BSD) test.
